# The Anterolateral Barrel Subfield Differs from the Posteromedial Barrel Subfield in the Morphology and Cell Density of Parvalbumin-Positive GABAergic Interneurons

**DOI:** 10.1523/ENEURO.0518-22.2024

**Published:** 2024-03-15

**Authors:** Naoki Shigematsu, Yuta Miyamoto, Shigeyuki Esumi, Takaichi Fukuda

**Affiliations:** Department of Anatomy and Neurobiology, Graduate School of Medical Sciences, Kumamoto University, Kumamoto 860-8556, Japan

**Keywords:** barrel, GABA, gap junction, parvalbumin, somatosensory

## Abstract

Layer 4 of the rodent somatosensory cortex has unitary structures called barrels that receive tactile information from individual vibrissae. Barrels in the anterolateral barrel subfield (ALBSF) are much smaller and have gained less attention than larger barrels in the posteromedial barrel subfield (PMBSF), though the former outnumber the latter. We compared the morphological features of barrels between the ALBSF and PMBSF in male mice using deformation-free tangential sections and confocal optical slice-based, precise reconstructions of barrels. The average volume of a single barrel in the ALBSF was 34.7% of that in the PMBSF, but the numerical density of parvalbumin (PV)-positive interneurons in the former was 1.49 times higher than that in the latter. Moreover, PV neuron density in septa was 2.08 times higher in the ALBSF than that in the PMBSF. The proportions of PV neuron number to both all neuron number and all GABAergic neuron number in the ALBSF were also higher than those in the PMBSF. Somata of PV neurons in barrels and septa in the ALBSF received 1.64 and 1.50 times more vesicular glutamate transporter Type 2–labeled boutons than those in the PMBSF, suggesting more potent feedforward inhibitory circuits in the ALBSF. The mode of connectivity through dendritic gap junctions among PV neurons also differed between the ALBSF and PMBSF. Clusters of smaller unitary structures containing a higher density of representative GABAergic interneurons with differential morphological features in the ALBSF suggest a division of functional roles in the two vibrissa–barrel systems, as has been demonstrated by behavioral studies.

## Significance Statement

The somatosensory cortex of rodents contains unique unitary structures called barrels that receive tactile information from individual vibrissae, a group of specialized thick hair around the nose. This vibrissa–barrel system enables animals to search and recognize surrounding objects. Barrels are located in two subfields, anterolateral barrel subfield (ALBSF) and posteromedial barrel subfield (PMBSF). The ALBSF contains much smaller barrels than the PMBSF and has gained little attention thus far despite the dense packing of many barrels. We show here that the ALBSF is further characterized by a higher density of parvalbumin-positive GABAergic interneurons, differential thalamocortical connectivity, and a distinctive mode of gap junction coupling. All these suggest different roles of the two vibrissa–barrel systems as reflected in behaviors.

## Introduction

Rodents use a special type of facial hair called whiskers or vibrissae to perceive surrounding objects. Sensory information acquired through whisker stimulations is diverse such as vibration frequency and phase and amplitude of whisker movement (Feldmeyer et al., 2013; Staiger and Petersen, 2021). Animals integrate such diverse information to understand their immediate environment. Signals from whiskers are sent to the primary somatosensory cortex through the trigeminal nucleus complex and two thalamic nuclei, namely, the ventroposterior medial thalamic nucleus (VPM) and the posterior medial nucleus (POm; Koralek et al., 1988; Chmielowska et al., 1989; Ma, 1991; Alloway, 2008; Feldmeyer, 2012). As in other sensory cortical areas that represent well-organized bodily or functional maps (Bednar and Wilson, 2016), layer 4 of the somatosensory cortical area receiving whisker-derived signals contains specialized maps that are composed of unitary structures called barrels. Each barrel consists of an accumulation of neurons targeted by thalamocortical axons that convey information from a single whisker. When viewed in tangential sections cut parallel to the cortical surface, barrels form a spatial pattern that preserves the arrangement of whiskers in the face (Woolsey and Van der Loos, 1970). The pattern can be visualized with not only the classical methods of Nissl staining and histochemistry for cytochrome oxidase (Wong-Riley and Welt, 1980) but also immunohistochemical staining for vesicular glutamate transporter Type 2 (VGluT2), where VGluT2 immunoreactivity visualizes the accumulation of thalamocortical axon terminals that contain this transporter protein (Fujiyama et al., 2001).

Most studies of barrels have been conducted in the posterior part of the primary somatosensory cortex containing larger barrels, which is termed posteromedial barrel subfield (PMBSF). In contrast, the anterolateral barrel subfield (ALBSF) is characterized by the packing of smaller barrels (Land and Simons, 1985; Jan et al., 2008). As to the corresponding area in the face, rodents have much shorter whiskers on their snout, especially around their mouths. These short whiskers are more densely packed and are called microvibrissae, whereas long whiskers are called macrovibrissae (Brecht et al., 1997). Previous behavioral studies using rats revealed that animals first use macrovibrissae to search and contact an object, followed by orienting to the object and the subsequent use of microvibrissae while bringing their snouts close to the object (Brecht et al., 1997; Grant et al., 2012). The movement of macrovibrissae such as palpation, brushing, tapping, and sweeping on an object is thought to be involved in environmental scanning and object cognition, whereas the use of microvibrissae has been related to the discrimination of objects (Brecht et al., 1997; Grant et al., 2012; Kuruppath et al., 2014), leading to the intriguing hypothesis that microvibrissae correspond to the fovea centralis of the retina in the visual system. However, the ALBSF has gained little attention so far, and information about it is limited.

Parvalbumin-immunopositive interneurons (PV neurons) constitute a major component of diverse neocortical GABAergic neurons, especially in layer 4 of the mouse cortex (Lee et al., 2010; Xu et al., 2010). PV neurons generate high-frequency trains of action potentials; thus, they are called fast-spiking (FS) neurons (Kawaguchi et al., 1987). Single PV neurons target the perisomatic membrane domain of hundreds of surrounding neurons and powerfully control the population activities. PV/FS neurons also establish dense mutual connections through both GABAergic synapses and dendrodendritic gap junctions (Galarreta and Hestrin, 1999; Gibson et al., 1999; Fukuda and Kosaka, 2000). PV neurons in the barrel cortex receive inputs from not only the local circuits but also brain-wide sources, especially other sensory cortices and the thalamus (Hafner et al., 2019). These prominent features lead to critical roles of PV neurons in a variety of cortical activities including feedback and feedforward inhibition, the regulation of the precise timing of action potential generation, network oscillation, and gain control in sensory responses (Sun et al., 2006; Atallah et al., 2012; Hu et al., 2014; Garcia-Junco-Clemente et al., 2018). Morphological features of PV neurons in the mouse PMBSF were extensively studied in previous studies (Staiger et al., 2009; Hafner et al., 2019) including the connectivity through dendritic gap junctions (Shigematsu et al., 2019).

In the present study, we focused on the morphological features of PV neurons in the ALBSF and compared them quantitatively with those in the PMBSF in male mice. We developed a new method to prepare deformation-free tangential sections to visualize the whole barrels in both subfields in a single section and revealed several differential features that suggest the functional segregation of the two vibrissa–barrel systems, as has been shown by accumulating evidence in behavioral studies.

## Materials and Methods

### Tissue preparation

All experiments and animal procedures were performed according to the Guide for the Care and Use of Laboratory Animals (National Institutes of Health Publication No. 86-23, revised 1987), and all protocols were approved by the Institutional Animal Care and Use Committee at our university. All efforts were made to minimize the number of animals used and their suffering. Under deep anesthesia with sodium pentobarbital (50 mg/kg, intraperitoneal injection), male C57 black 6J mice (RRID:IMSR_JAX:000664, 20–25 g, 7–8 weeks old) were perfused from the ascending aorta with 4% paraformaldehyde in 0.1 M phosphate buffer (pH 7.2–7.4). After perfusion with the fixative, brains were gently removed from the skull and stored overnight in the fixative at 4°C. The next day, the fixative was replaced by 0.01 M phosphate-buffered saline (PBS, pH 7.2–7.4) that contained 0.1% sodium azide.

### Preparation of deformation-free tangential sections

**Figure 1.**
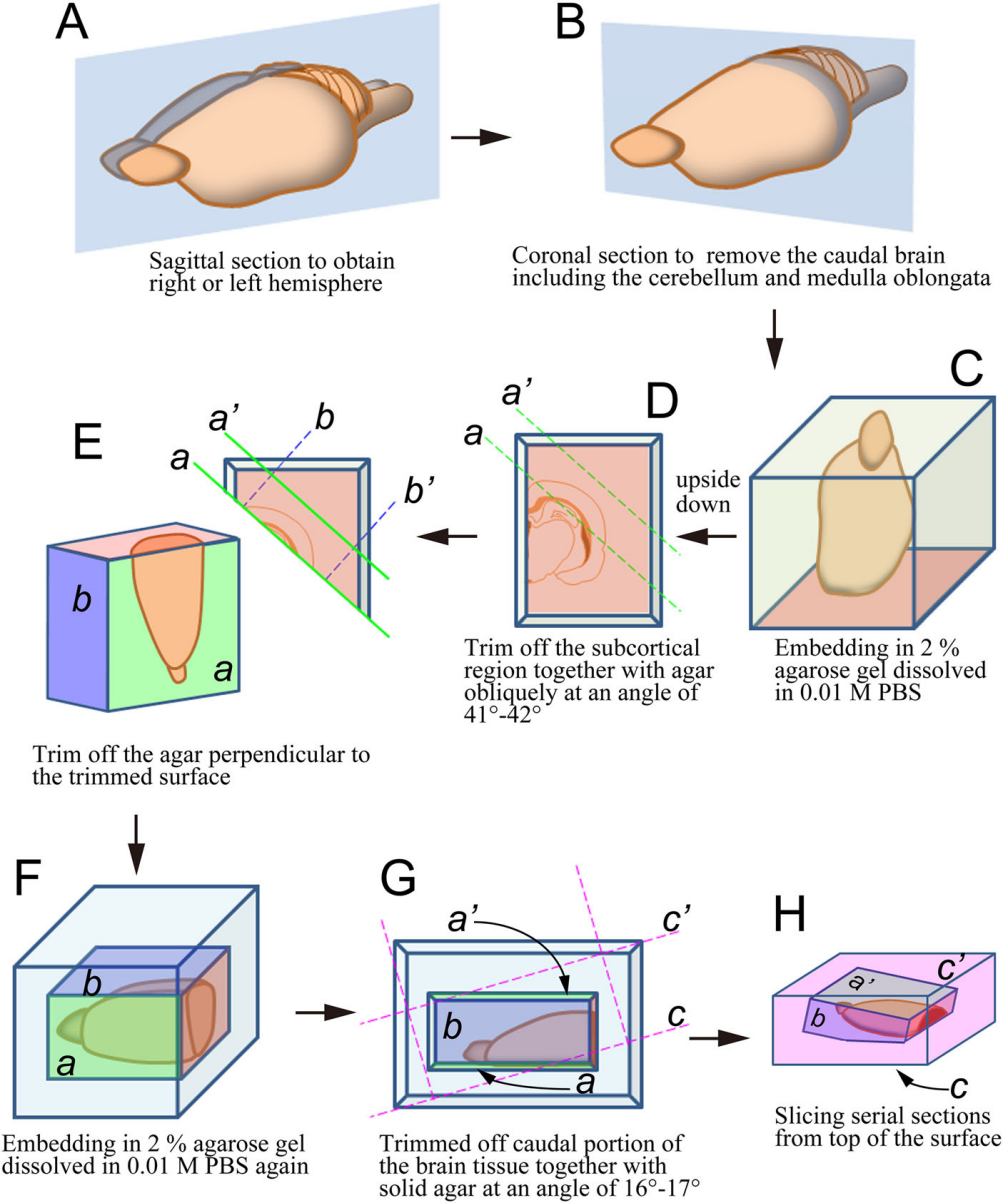
Preparation of deformation-free tangential sections. A brain hemisphere is embedded in agarose gel, trimmed at appropriate angles twice, and then attached to the boat of a vibrating microtome. More details are described in Materials and Methods. The barrel cortex becomes horizontal after these procedures. Serial tangential sections expose the entire array of barrels in both the ALBSF and PMBSF in a single section as shown in Figure 2.

First, we performed a coronal cut of a brain hemisphere to remove the caudal brain including the most caudal part of the hemisphere, cerebellum, pons, and medulla oblongata (Fig. 1*A,B*). The remaining brain tissue was embedded in 2% agarose gel dissolved in 0.01 M PBS (Fig. 1*C*) with the caudal cut surface set at the bottom of the mold. After hardening the gel, the embedded tissue was placed upside down, and then, the subcortical region together with agar was trimmed off obliquely at an angle of 41–42° relative to the midsagittal plane to make a new plane *a* (Fig. 1*D*). Agar on the lateral side was cut to make a plane *a*′ parallel to plane *a*. The remaining agar was trimmed off at right angles to *a* and *a*′ to expose a new surface (plane b) dorsally and another new surface (plane *b*′) ventrally, resulting in a right rectangular prism containing the brain (Fig. 1*E*). Then, the specimen was rotated to stand planes *a* and *a*′ perpendicular to the lab bench, while plane *b* was at the topside (Fig. 1*F*). The whole specimen was further embedded in agar to obtain a rectangular prism of one size larger. The final trimming was performed by cutting through a newly added agar with an angle of 16–17° to plane *a* to obtain a new rectangular prism (Fig. 1*G*). The newly exposed surface, designated plane *c* in Figure 1*G*, was placed at the bottom side and attached to the boat of the vibrating microtome (TTK-3000, Dosaka) so that the brain surface covering the barrel cortex became horizontal (Fig. 1*H*). Serial 40-µm-thick sections were cut from the cortical surface using the vibrating microtome.

### Immunohistochemistry

Serial tangential sections were incubated in PBS containing 12.5% (w/w) and 25% (w/w) sucrose, each for 1 d, at 20°C. Then, sections were subjected to rapid freezing in the vapor of liquid N_2_ and rapid thawing in PBS containing 25% sucrose. After blocking in PBS containing 1% bovine serum albumin, 0.3% Triton X-100, and 0.1% sodium azide, sections were processed for immunohistochemistry using one of the three sets of antibody mixtures: (1) a mixture of rabbit antibody against PV (Swant; catalog #PV 25 RRID:AB_10000344; dilution 1:5,000) and guinea pig antibody against VGluT2 (Frontier Institute; catalog #VGluT2-GP RRID:AB_2571621; dilution 1:500); (2) a mixture of rabbit antibody against PV, guinea pig antibody against VGluT2, and mouse monoclonal antibody against NeuN (Merck Millipore; catalog #MAB377, RRID:AB_2298772; dilution 1:500); and (3) a mixture of mouse monoclonal antibody against PV (Swant; catalog #PV 25 RRID:AB 10000344; dilution 1:5,000), guinea pig anti-VGluT2 antibody (dilution 1:500), and rabbit antibody against connexin 36 (Cx36; NCX-1; Shigematsu et al., 2019; dilution 1:500). Primary antibodies were dissolved in the blocking solution for 7 d at 20°C. The long incubation period with the primary antibodies was essential to improve the penetration of the antibodies into the deep part of the 40-µm-thick sections and thus to obtain confocal images of constant and sufficient quality throughout the depth of each section.

After several rinses in PBS, sections were incubated overnight at 20°C with secondary antibodies that were appropriately combined in each of the three sets of primary antibody mixtures described above: for mixture 1, Alexa 488–conjugated donkey antirabbit IgG (Molecular Probes; catalog #A-21206 RRID:AB_141708; dilution 1:250) and Cy3-conjugated antiguinea pig IgG (Jackson ImmunoResearch; catalog #706-166-148 RRID:AB_2340461; dilution 1:250); for mixture 2, biotinylated donkey antirabbit IgG (Jackson ImmunoResearch; catalog #715-065-151 RRID:AB_2340785; dilution 1:250), followed by Alexa 647–conjugated streptavidin (Jackson ImmunoResearch; catalog #016-600-084 RRID:AB_2341101; dilution 1:250), donkey Cy3-conjugated antiguinea pig IgG (dilution 1:250), and Alexa 488–conjugated antimouse IgG (Jackson ImmunoResearch; catalog #715-545-150 RRID:AB_2340846; dilution 1:250); and for mixture 3, Alexa 488–conjugated donkey antimouse IgG (Jackson ImmunoResearch; catalog #711-545-151; dilution 1:250), Alexa 647–conjugated antiguinea pig IgG (Jackson ImmunoResearch; catalog #706-605-148 RRID:AB_2340476; dilution 1:250), and Rhodamine Red–conjugated donkey antirabbit IgG (Jackson ImmunoResearch; catalog #711-295-152; dilution 1:100). Sections were mounted in VECTASHIELD antifading medium (Vector Laboratories; catalog #H-1000 RRID:AB_2336789) and examined using a confocal laser scanning light microscope (CLSM; C2, Nikon) equipped with 10× (Plan Fluor, NA = 0.30), 20× (Plan Fluor, NA = 0.50), and 60× (Plan Apo VC, NA = 1.40) oil objectives. Single laser beams (488, 543, and 633 nm) and filter sets of BA 515/30 and BA 590/50 and 650 LP were alternately used to collect images for different fluorescent signals. The size of each frame was 1,024 × 1,024 pixels, and images of optical slices were acquired from the section surface to the bottom at the preset optical step size (10×, 3.25 µm; 20×, 1.1 µm; 60×, 0.15 µm) in each of the 5–6 serial 40-µm-thick sections that covered the entire structure of barrels.

### 3D reconstructions of barrels for quantitative analysis

**Figure 2.**
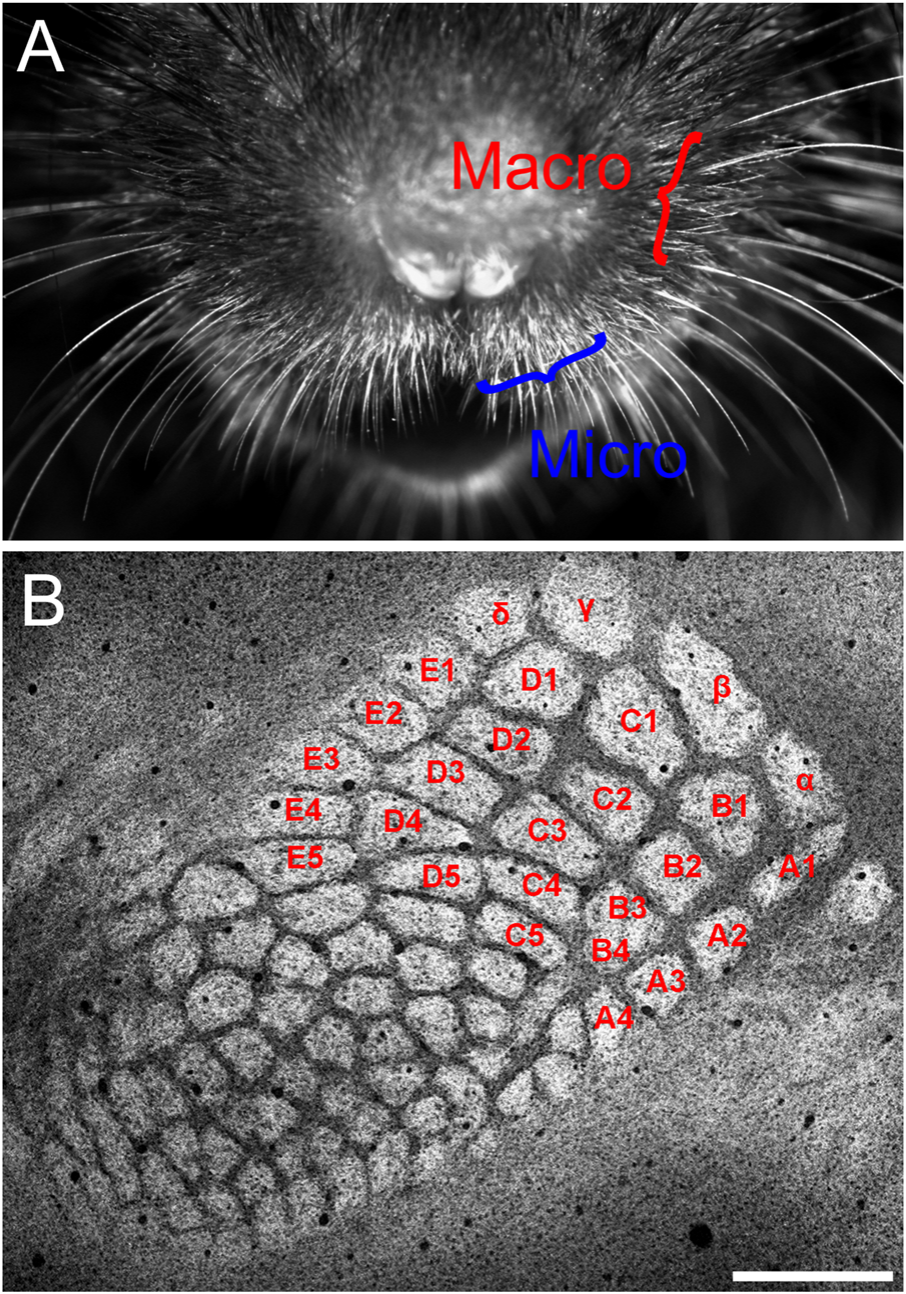
The arrangement of vibrissae and barrels. ***A***, Front view of micro- and macrovibrissae. ***B***, Tangential section through the mouse somatosensory cortex immunolabeled for VGluT2, showing both the ALBSF and PMBSF in a single section. Names of barrels in the PMBSF are labeled to identify individual barrels. Scale bar, 500 µm.

**Figure 3.**
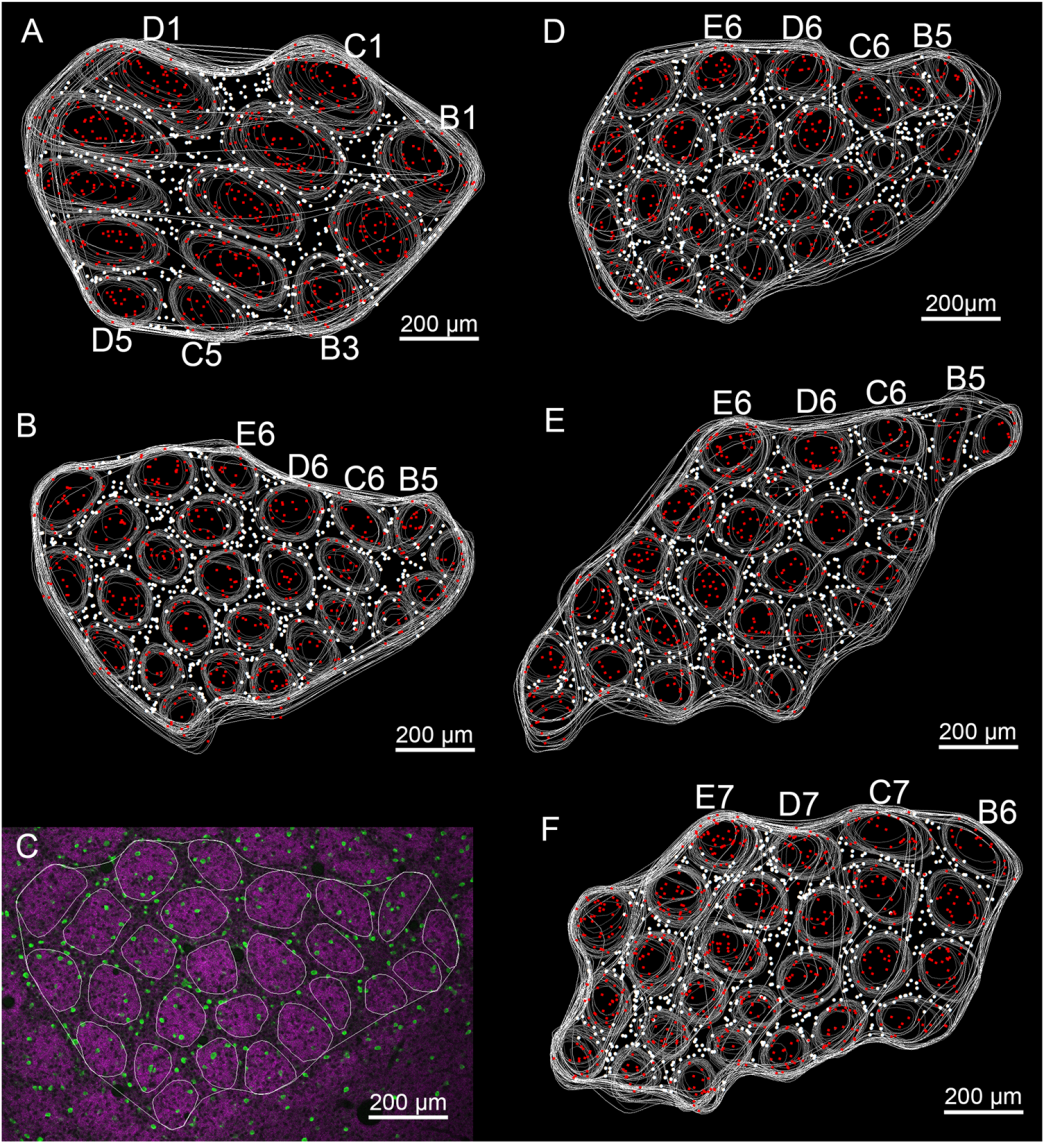
Traces of barrels in tangential sections cut from different animals and used for quantitative analyses. The red and white dots indicate the positions of PV neurons located inside barrels and septa, respectively. ***A***, Outlines of 13 barrels (B1–B3, C1–C5, and D1–D5) in the PMBSF were traced in individual single optical slices in CLSM images as shown for ALBSF in ***C***. Then, all traces outlining each barrel at different depths from the brain surface were projected onto one plane. The outermost contour surrounding the cluster of barrels at each depth from the brain surface is used to measure the area of septa that intervened barrel clusters at each depth of the optical slice, by subtracting the sum of barrel areas from the area inside the outermost contour. ***B***, ***D–F***, Outlines of 25–26 barrels in the ALBSF. Selections of barrels started from B5, C6, D6, and E6 and extended anterolaterally under the rule of keeping direct contact with one another to ensure the objective and unbiased selection method. In ***F***, selection started from B6, C7, D7, and E7 due to a slight difference in the angle of tangential sectioning that resulted in irregular shapes of B5 and C6–E6 barrels. An example of tracing in a single optical slice in the ALBSF is demonstrated in ***C***, where immunoreactivities for PV and VGluT2 are shown in green and magenta, respectively. The contour of each barrel is clearly delineated by the accumulation of VGluT2-positive boutons in barrels. Note that barrels in the ALBSF are arrayed rather irregularly with different shapes and sizes.

The two barrel subfields were defined according to the relative position (Fig. 2*B*): one subfield containing the 27 posteromedial barrels (α–δ, A1–A4, B1–B4, C1–C5, D1–D5, E1–E5) that were named in conformity with the established usage and the other consisting of the remaining barrels located at more anterolateral position. For convenience of quantification, the former was defined as PMBSF and the latter ALBSF in the present analysis. In each hemisphere of five animals, 13 barrels in the PMBSF and 25–26 barrels in the ALBSF were selected for quantitative analysis (Fig. 3). The former consisted of B1–B3, C1–C5, and D1–D5 (Fig. 3*A*). The latter consisted of barrels clustering at the following position (Fig. 3*B–E*), beginning from B5, C6, D6, and E6, extending anterolaterally, and forming a sampling area where 25–26 barrels were in direct contact with one another. This sample size, 25–26 barrels in the ALBSF, was determined so that the area occupied by these barrels became comparable to the area occupied by 13 barrels in the PMBSF. In one animal, selection of ALBSF barrels started from B6, C7, D7, and E7 (Fig. 3*F*), followed by the same procedure as in other four animals: a slight difference in the angle of tangential sectioning resulted in rather irregular shapes of B5, C6, D6, and E6 barrels in this animal. During observations using confocal optical slices, we found that each barrel had an irregular shape rather than a simple rectangular or oval shape and that each barrel should be reconstructed according to its original shape for a detailed quantitative analysis; otherwise, it became difficult to determine the precise position of individual neurons whether they were located inside a barrel or septa. Therefore, barrels selected from the PMBSF and ALBSF as above were reconstructed by tracing the contour of the accumulation of VGluT2-positive boutons in each optical slice (Fig. 3*C*) using the confocal module of the computer-assisted neuron tracing system Neurolucida (MBF Bioscience; RRID:SCR_001775). Images for tracing of contours were taken with a 10× objective at an original step size of 3.25 µm: tracing for reconstruction was made in every other optical slice, from the top to the bottom of each 40-μm-thick section. Because barrels spanned several 40-μm-thick tangential sections, traces of individual barrels were acquired from neighboring 4–5 sections and were combined to reconstruct the whole structures of barrels (Fig. 3*A,B,D–F*) using the procedure of precisely matching the surfaces of neighboring sections using cut ends of blood capillaries as landmarks (Kosaka et al., 1985). The volume of each barrel was calculated by measuring the area of the barrel that was observable in each optical slice, summing the areas of that barrel found in a series of optical slices, and then multiplying the summated areas by the distance between each trace (6.50 µm). The obtained volume was corrected for a refractory index of glass (1.5) but was not corrected for shrinkage because we confirmed slightness of shrinkage in the present materials that were not processed for alcohol dehydration. Moreover, sections used for comparison between the two barrel subfields were always processed simultaneously. Therefore, slight shrinkage, if present, was thought to have the same effect on the size of barrels in the two subfields. We identified PV-positive somata in each optical slice using a 20× objective and marked their locations inside or outside the barrels using different symbols (Fig. 3*A,B,D–F*). The total number of PV neurons in each barrel was divided by the barrel volume to calculate the numerical density of PV neurons.

The numerical density of PV neurons in the septa was analyzed in the defined area that covered the reconstructed barrels in the PMBSF and ALBSF (Fig. 3). The volume of the analyzed portion of septa was calculated as follows: (1) the whole area covering the sampled barrels (13 barrels in the PMBSF and 25–26 barrels in the ALBSF) plus intervening septa was measured in each optical slice by tracing a contour surrounding all the sampled barrels and septa (Fig. 3*C*) using Neurolucida; (2) the area occupied by the septa, which was viewed as one continuous structure inside the abovementioned contour, was obtained by subtracting the sum of areas of sampled barrels from the whole area described in (1); and (3) the volume of the septa spanning several 40-μm-thick tangential sections, viewed as a single 3D stricture, was calculated according to the same procedures that were described for calculation of each barrel volume. The numerical density of PV neurons per unit volume was calculated by dividing the cell number by the volume of the septa.

### Analysis of the proportion of PV neuron number to total neuron number

Serial 40-µm-thick sections were cut in the coronal direction from the somatosensory cortex of five mice and were triple-immunostained for PV, VGluT2, and NeuN as described above. Two sections at different rostrocaudal positions were selected from each animal, one at the bregma and the other located 1.7 mm caudal to the bregma, to detect anterolateral and posteromedial barrels, respectively. Barrels were visualized by an accumulation of VGluT2-positive boutons, and their images were acquired using a 20× objective in 1,024 × 1,024 pixels with a step size of 1.1 µm. Three neighboring barrels in the ALBSF (*n* = 5 animals) and three (*n* = 4 animals) to four (*n* = 1 animal) neighboring barrels in the PMBSF were reconstructed by tracing the contour in every other optical slice. After the reconstruction of barrels that were contained in single sections, the numbers of PV- and NeuN-positive cells were counted in barrels using Neurolucida on the basis of the stereological procedure “disector” (Sterio, 1984). The proportions in the septa were also calculated in a way similar to that in barrels. The septal areas used for analysis were those intervening the 3–4 neighboring barrels that were used for the analysis of PV/NeuN proportions.

### Analysis of the proportion of PV neuron number to the total number of GABAergic neurons

Serial 40-µm-thick sections were cut in the coronal direction from the somatosensory cortex of GAD67-green fluorescent protein (GFP) knock-in mice (Tamamaki et al., 2003; *n* = 5 mice) and were triple-immunostained for PV, VGluT2, and GFP. Two sections at different rostrocaudal positions were selected from each animal, one at the bregma and the other located 1.7 mm caudal to the bregma, to detect anterolateral and posteromedial barrels, respectively. Detection of GFP-expressing cells was performed using rat anti-GFP antibody (Nacalai Tesque; catalog#04404-84, RRID:AB_10013361, dilution 1:1,000) followed by labeling with Alexa 488–conjugated antirat IgG (Jackson ImmunoResearch; catalog #712-545-153, RRID:AB_2340684; dilution 1:250); labeling for PV and VGluT2 was performed simultaneously with GFP labeling by the same method as in triple labeling for PV, VGLuT2, and NeuN described above. Three barrels and intervening septal areas were reconstructed in each rostral and caudal section in each mouse by tracing the contour in every other optical slice using CLSM images acquired with a 20× objective, followed by cell counting and calculation of PV/GAD67 proportion as in the measurement of PV/NeuN proportion.

### 3D reconstructions of PV neurons in the ALBSF

To analyze the detailed morphology of PV neurons in the ALBSF, we reconstructed two neighboring barrels in coronal sections that were triple-immunostained for PV, VGluT2, and Cx36 (*n* = 5 animals). Using a 20× objective, barrel boundaries were traced in optical slices 2.2 µm apart in the *z*-direction as above. This tracing procedure was accomplished throughout two serial 40-µm-thick sections that almost contained the entire structure of the barrels. High-resolution images of somata and dendrites of PV neurons were acquired using a 60× objective and a further magnification rate of 1.5 in the CLSM setting, followed by tracing with Neurolucida. During this tracing procedure, sites of contacts between VGluT2-positive boutons and PV-positive soma and dendrites were detected using a 60× objective and recorded using Neurolucida. In addition, dendrodendritic connections through neuronal gap junctions were also recorded, using Neurolucida, at the positions where Cx36-positive puncta were detected between PV-positive dendrites that made contact with each other (Fig. 9*C*).

### Data analysis

All images in the figures demonstrated were prepared using ImageJ 1.44 (NIH: http://imagej.nih.gov/ij/) and Adobe Photoshop CS5 software (Adobe Systems).

Statistical examinations were performed using GraphPad Prism 9 (GraphPad Software). Quantitative data are presented as the mean ± standard deviation (SD). The significance level was set at a *p* value of <0.05 when a single test using the Mann–Whitney *U* test was performed under one null hypothesis. When multiple comparisons were made among a family of tests under the same null hypothesis, appropriate levels of significance were assessed using two methods: one was Bonferroni’s (Bf) correction of α level and the other was the Benjamini–Hochberg (B–H) procedure, setting the false discovery rate at 0.05 (Benjamini and Hochberg, 1995).

## Results

### Visualization of barrels in deformation-free tangential sections

Previous studies have shown that the pattern of methodical arrangement of mouse vibrissae is held in the pattern of barrels located in layer 4 of the primary somatosensory cortex (Woolsey and Van der Loos, 1970). In many studies, cortical tissues having a convex surface were flattened before cutting tangential sections to visualize as many barrels as possible in single sections. However, this procedure inevitably results in the deformation of the three-dimensional structure of barrels, especially at the periphery of flattened tissue, which will hamper precise quantitative analysis. Thus, we tried to find an optimal plane to cut tangential sections in which the arrangement of barrels could be confirmed over the entire barrel field in a single section without pretreatments for flattening (Figs. 1, 2*B*). According to the standard nomenclature, 27 barrels located in the posteromedial position were identified in most animals as α–δ, A1–A4, B1–B4, C1–C5, D1–D5, and E1–E5. Among them, the arrangement of 23 barrels named by the English alphabet was composed of five rows (A–E) and 4–5 arcs, the latter corresponding to the curves drawn along barrels of the same number, e.g., A1–E1. At the posteromedial border, there were four large barrels (α–δ) that have been conventionally termed straddlers. They were located in the positions between two rows in the first arc, such as α between A1 and B1, β between B1 and C1, and so on. The arrangement of these barrels in the posteromedial position was basically similar with slight differences across animals, as described in previous studies (Woolsey and Van der Loos, 1970; Jan et al., 2008). In contrast, the remaining barrels located anterolateral to the above 27 barrels became gradually smaller and lost the regular “row and arc” pattern; barrels in neighboring rows intermingled with each other. Moreover, the arrangement of anterolateral barrels differed from animal to animal (Figs. 2*B*, 3*B–F*). Thus, it was difficult to allocate consistent numbers to barrels in the anterolateral position. This raises an issue of how objectively the barrel field can be divided into anterolateral and posteromedial subfields. In the present study, we simply defined two subfields according to the relative position as described in Materials and Methods; the area consisting of the posterolateral 27 barrels mentioned above was defined as the PMBSF and the remaining region as the ALBSF.

### Comparisons of the barrel size and numerical density of PV neurons between ALBSF and PMBSF

**Figure 4.**
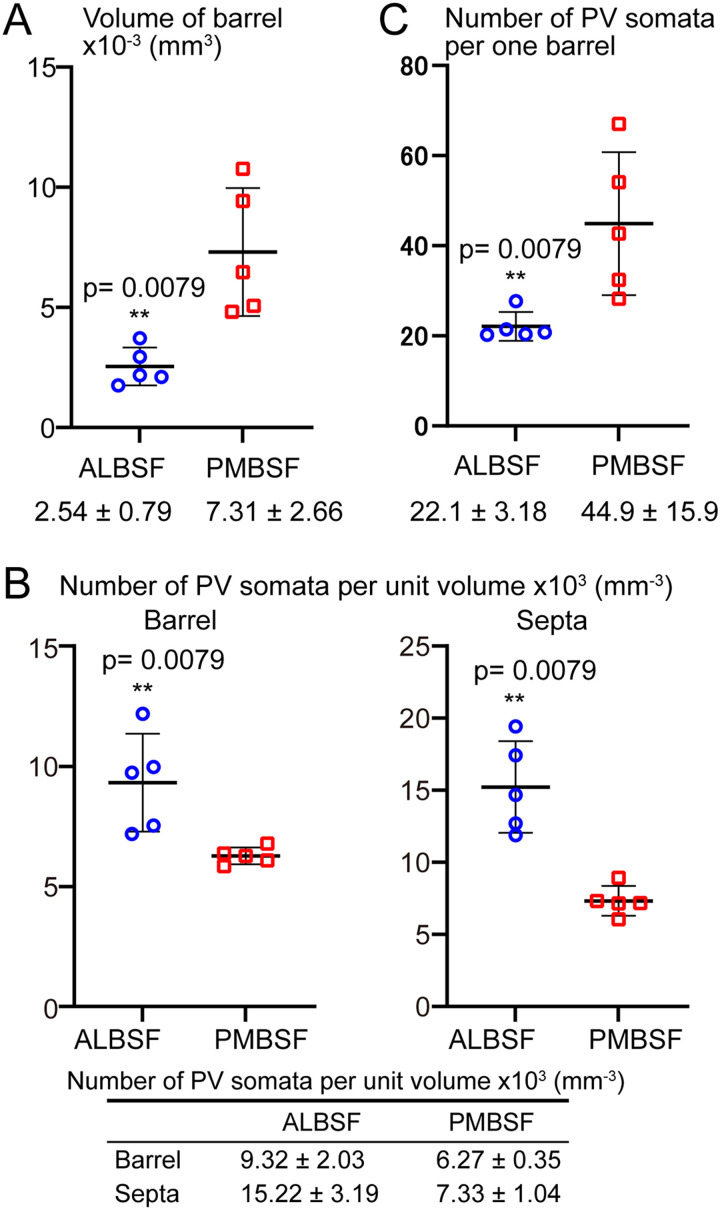
Comparisons of the size of barrels and of the numerical data of PV neurons between the ALBSF and PMBSF. ***A***, Averaged volume of single barrels measured in the ALBSF and PMBSF (*n* = 5 mice each). ***B***, Density of PV-positive somata (number per unit volume) located in barrels (left) and septa (right) (*n* = 5 mice each). ***C***, Number of PV-positive somata in single barrels (*n* = 5 mice each). Statistical analysis is shown in Table 1.

In each animal, 13 barrels (B1–B3, C1–C5, D1–D5) in the PMBSF and 25–26 barrels in the ALBSF were objectively selected for quantitative analysis (Fig. 3; see Materials and Methods). The average volume of individual barrels (Fig. 4*A*) was calculated and compared between the two barrel subfields (*n* = 5 mice each). The average volume of barrels in the ALBSF was 34.7% of that in the PMBSF, and the difference was statistically significant (*p* = 0.0079 in Mann–Whitney *U* test).

Next, the number of PV neurons in each barrel was counted and divided by the volume of that barrel to obtain the density of PV neurons (number per unit volume) in single barrels in both the PMBSF and ALBSF. These densities were averaged in each animal, followed by further averaging in five animals (Fig. 4*B*, left). The density in the ALBSF was 1.49 times higher than that in the PMBSF, and this difference was statistically significant (*p* = 0.0079 in Mann–Whitney *U* test, followed by both Bf correction and B–H procedure, Table 1).

**Table 1.** Statistical analysis of multiple comparisons

Figure	Pair for comparison	*i*	pi		FDR × i/N	FDR	B-H test	*a* in Bonferroni’s	Bf test
4*B*	PMBSF barrel vs septa	4	0.0952	>	0.05	0.05	n.s.	0.0125	n.s.
	ALBSF barrel vs septa	3	0.0159	<	0.0375	0.05	Significant	0.0125	n.s.
	ALBSF septa vs PMBSF septa	2	0.0079	<	0.025	0.05	Significant	0.0125	Significant
	ALBSF barrel vs PMBSF barrel	1	0.0079	<	0.0125	0.05	Significant	0.0125	Significant
5*C*	ALBSF septa vs PMBSF septa	2	0.0159	<	0.05	0.05	Significant	0.025	Significant
	ALBSF barrel vs PMBSF barrel	1	0.0079	<	0.025	0.05	Significant	0.025	Significant
6*C*	ALBSF septa vs PMBSF septa	2	0.0159	<	0.05	0.05	Significant	0.025	Significant
	ALBSF barrel vs PMBSF barrel	1	0.0079	<	0.025	0.05	Significant	0.025	Significant
8*D*	ALBSF Type 3 vs PMBSF Type 3	2	0.0194	<	0.05	0.05	Significant	0.025	Significant
	ALBSF Type 2 vs PMBSF Type 2	1	<0.0001	<	0.025	0.05	Significant	0.025	Significant
9*B*	ALBSF Type 3 vs PMBSF Type 3	2	0.1017	>	0.05	0.05	n.s.	0.025	n.s.
	ALBSF Type 2 vs PMBSF Type 2	1	0.0037	<	0.025	0.05	Significant	0.025	Significant

*i*, the order of *p* value; pi, *p* value in Mann–Whitney *U* test for each comparison; *N*, the number of multiple comparisons; FDR, false discovery rate; B–H test, Benjamini–Hochberg test; Bf test, Bonferroni’s test.

We further compared the density of PV neurons in the septa between the ALBSF and BMBSF (Fig. 4*B*, right). In each animal, the density was calculated in a single continuous septal space that intervened a cluster of barrels used for the above analysis, consisting of 13 barrels in the PMBSF and 25–26 barrels in the ALBSF (see Materials and Methods). The density measured in five animals was averaged and compared between the ALBSF and PMBSF. The density in the ALBSF was 2.08 times higher than that in the PMBSF with a statistically significant difference (*p* = 0.0079 in Mann–Whitney *U* test, followed by both Bf correction and B–H procedure, Table 1).

The PV neuron density was also compared between the barrel and septa in each barrel subfield (Fig. 4*B*). In the PMBSF, there was no statistical difference in the density of PV neurons between the barrel and septa (*p* = 0.0952 in Mann–Whitney *U* test, followed by both Bf correction and B–H procedure, Table 1), which is consistent to a previous analysis (Almási et al., 2019). In contrast, the PV neuron density in the ALBSF barrel was lower than that in the ALBSF septa (*p* = 0.0159 in Mann–Whitney *U* test, which was assessed as significant by B–H procedure but not in Bf correction, Table 1).

During the analysis of PV neuron density, the number of PV neurons contained in single barrels was counted and averaged (Fig. 4*C*). The number in the ALBSF was nearly half (49.2%) the number in the PMBSF with a statistically significant difference (*p* = 0.0079 in Mann–Whitney *U* test).

### Comparison of the proportions of PV neuron number to total neuron number between ALBSF and PMBSF

**Figure 5.**
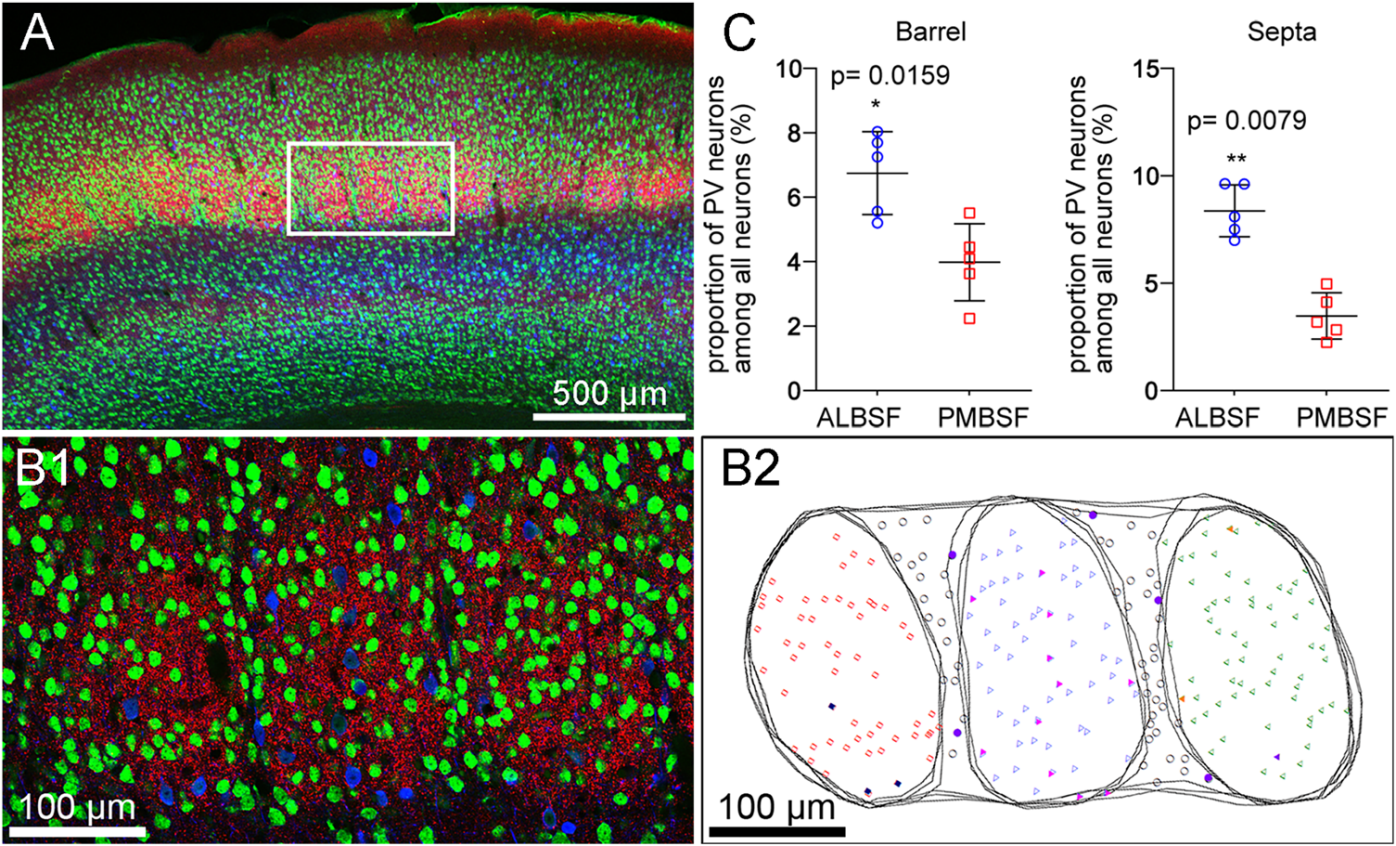
Comparison of the proportion of the number of PV neurons to the number of all neurons between the ALBSF and the PMBSF. ***A***, Low magnification (10×) CLSM image of a coronal section containing the ALBSF, labeled for NeuN (green), VGluT2 (red), and PV (blue). The boxed area, enlarged in ***B1***, contains three barrels in the ALBSF. ***B1***, Higher magnification (20×) of the boxed area in ***A***, shown in a projection image of six optical slices corresponding to 9.9 µm in corrected thickness. Accumulation of VGluT2-immunoreactive boutons visualizes three barrels. ***B2***, The contours of three barrels and laminar borders in ***B1*** are traced in three successive optical slices. NeuN-positive neurons are marked by open symbols of different types, whereas PV neurons are marked by filled symbols of different types. ***C***, Comparisons of the proportion of PV neuron number to all neuron number between the ALBSF (blue) and PMBSF (red) in both barrels (left) and septa (right). The proportions of PV neurons to all neurons in barrels were 6.75 ± 1.15% in the ALBSF (*n* = 5 animals, 3 neighboring barrels in 1 coronal section in each animal) and 3.99 ± 1.07% in the PMBSF (*n* = 5 animals, 3–4 neighboring barrels in 1 coronal section in each animal), respectively. The proportion in the septa was 8.37 ± 1.08% in the ALBSF (*n* = 5 animals, 1 section containing 2 septal regions intervening 3 barrels in each animal) and 3.47 ± 0.96% in the PMBSF (*n* = 5 animals, 2–3 septal regions intervening 3–4 neighboring barrels in 1 coronal section in each animal). Statistical analysis is shown in Table 1.

We further compared the proportion of the number of PV neurons to the number of all neurons contained in a single barrel between the two barrel subfields, because the higher density of PV neurons per unit volume of the ALBSF might simply reflect the higher density of all neurons inside anterolateral barrels without a difference in the proportion of PV neuron number to all neuron number. The proportion was calculated using CLSM images of triple immunofluorescence for PV, NeuN, and VGluT2 in coronal sections (Fig. 5), where NeuN-positive cells were assumed to represent most, if not all, neurons located in the respective regions. The proportion in barrels in the ALBSF was 1.69 times higher than that in the PMBSF, with a statistically significant difference (Fig. 5*C*, *p* = 0.0079 in Mann–Whitney *U* test, followed by both Bf correction and B–H procedure, Table 1). The proportion in septa in the ALBSF was 2.41 times higher than that in the PMBSF with a statistically significant difference (Fig. 5*C*, *p* = 0.0159 in Mann–Whitney test, followed by both Bf correction and B–H procedure, Table 1).

Measurements of these proportions further enabled us to estimate the total number of neurons that were contained in single barrels. As described above, we counted the numbers of PV neurons in single barrels and averaged them in both the ALBSF and PMBSF (Fig. 4*C*). Dividing these averages by the proportion of the number of PV neurons to the total neuron number yielded the estimated number of neurons in single barrels, which were 327 and 1,125 in the ALBSF and PMBSF, respectively. Thus, the estimated number of neurons in single barrels in the ALBSF was 29.0% of that in the PMBSF.

Because the C2 barrel in the mouse PMBSF has been repeatedly chosen for the analysis of barrel function in vivo (Sofroniew et al., 2015; Lefort and Petersen, 2017; van der Bourg et al., 2017), we estimated the total number of neurons in the C2 barrel using the measured number of PV neurons in C2, which was obtained during the above analysis covering 13 barrels in the PMBSF. The average number of PV neurons in C2 was 68.0 ± 15.2 (*n* = 5). Dividing this value by the average proportion of PV neuron number to total neuron number (3.99%; Fig. 5*C*) yielded 1,704 neurons in a single C2 barrel. We also measured the average volume of the C2 barrel which was 10.2 ± 3.2 × 10^−3^ mm^3^. Thus, the density of PV neurons (number per unit volume) in C2 was calculated to be 6.67; this value was comparable to the averaged density of 13 barrels in the PMBSF (6.27 ± 0.35; Fig. 4*B*; *n* = 5 animals).

### Comparison of the proportions of the number of PV neurons to the number of GABAergic neurons between the ALBSF and PMBSF

**Figure 6.**
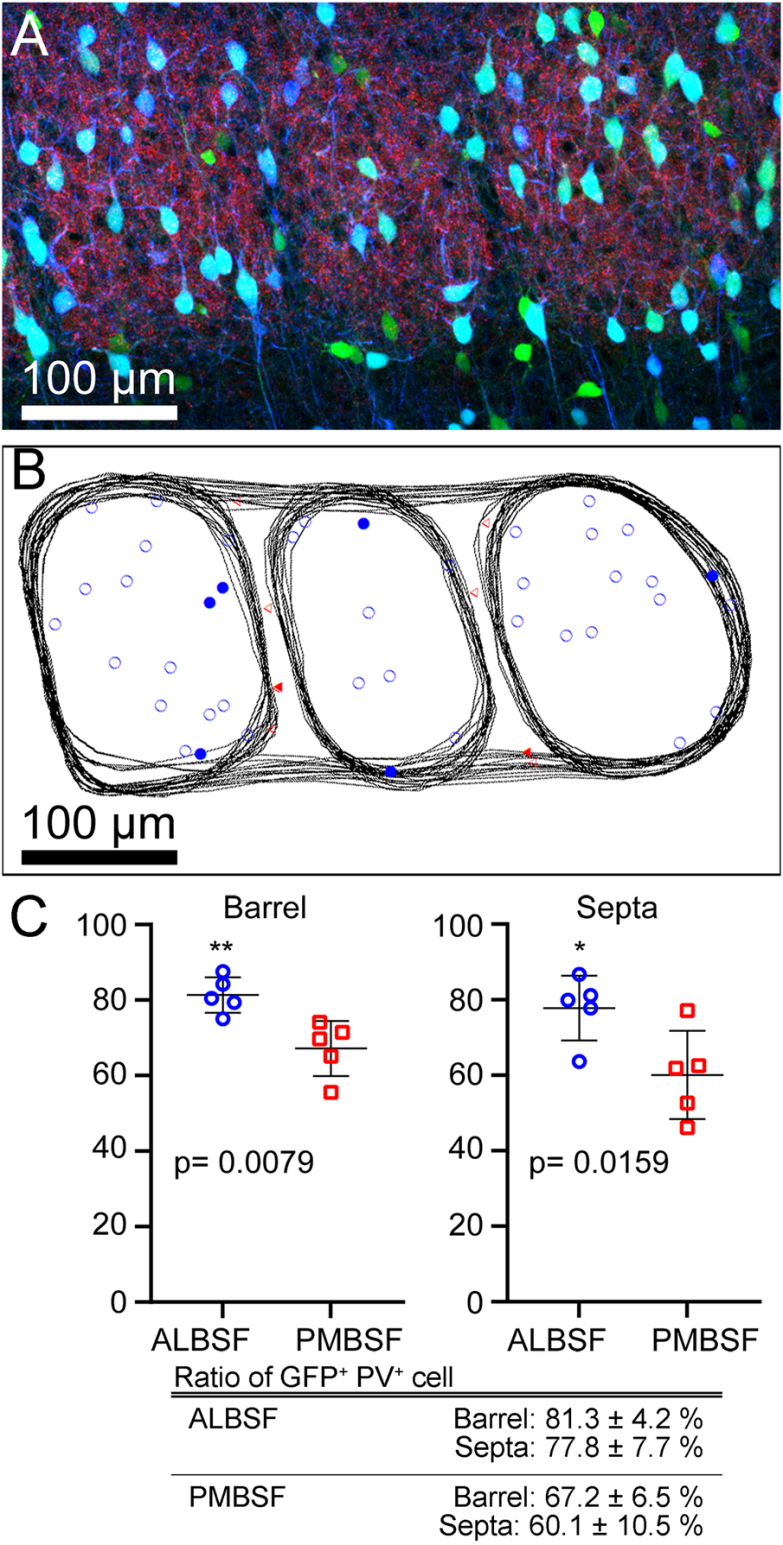
Comparison of the proportion of the number of PV neurons to the number of all GABAergic neurons between the ALBSF and the PMBSF. ***A***, CLSM image of a coronal section containing the ALBSF, labeled for GAD67-GFP (green), VGluT2 (red), and PV (blue). All optical slices in a single section, acquired through a 20× objective, were projected onto a plane. The accumulation of VGluT2-immunoreactive boutons visualizes three barrels. ***B***, The contours of three barrels and laminar borders in ***A*** are traced in all successive optical slices contained in a single section. GAD67/PV-double–positive neurons are marked by open symbols of different types, whereas PV-negative/GAD67-positive neurons are marked by closed symbols. On a basis of the principle of disector analysis, some neurons recognizable in ***A*** were not selected for counting. ***C***, Comparison of the proportion of PV neurons to all GABAergic neurons (*bottom*) between the ALBSF (blue) and PMBSF (red) in both barrels (left) and septa (right). Statistical analysis is shown in Table 1.

Next, we compared the proportion of the number of PV neurons to the number of all GABAergic interneurons in a single barrel between the two barrel subfields (Fig. 6), using GAD67-GFP knock-in mice (Tamamaki et al., 2003). The proportions of PV neurons to GFP-positive cells in barrels in the ALBSF was 1.20 times higher than that in the PMBSF, with a statistically significant difference (*p* = 0.0079 in Mann–Whitney *U* test, followed by both Bf correction and B–H procedure, Table 1). The proportion of PV neurons in septa in the ALBSF was 1.29 times higher than that in the PMBSF, with a statistically significant difference (*p* = 0.0159 in Mann–Whitney *U* test, followed by both Bf correction and B–H procedure, Table 1).

### Morphological features of PV neurons in the ALBSF

**Figure 7.**
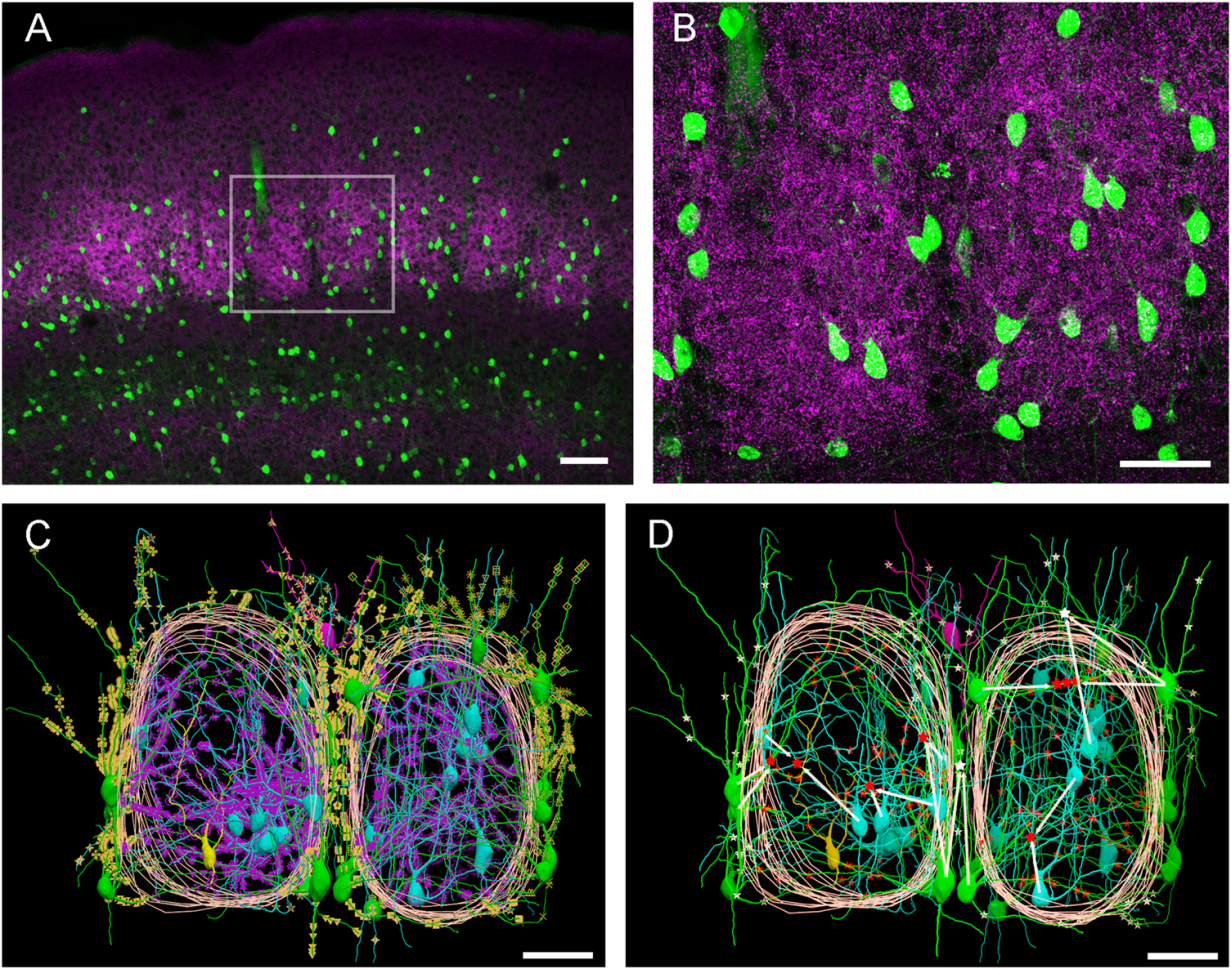
Distributions of thalamocortical inputs and gap junctions along dendrites of PV neurons in the ALBSF. ***A***, Low magnification CLMS image of PV (green) and VGluT2 (magenta) immunoreactivities in the rostral part of the mouse somatosensory cortex that contains the ALBSF. ***B***, Higher magnification of the boxed area in ***A***, showing two barrels in the ALBSF. A projection image of multiple optical slices. ***C***, Reconstructions of both PV neurons located in and around the two barrels and VGluT2-labeled boutons on dendrites in the ALBSF barrels shown in ***B***. Reconstructions were made by combining tracings from three successive sections that spanned the barrels. In total, 34 PV neurons were reconstructed and classified into four subtypes according to the position of soma and dendrites: Type 1 (in-barrel type, yellow), soma and all dendrites are confined within a single barrel; Type 2 (trans-barrel type, cyan), soma is located inside a barrel, whereas dendrites cross the barrel border toward the outside of the home barrel; Type 3 (trans-septa type, green), soma is located in the septa, whereas at least one dendrite penetrates into the adjoining barrel; and Type 4 (in-septa, magenta), the soma and all dendrites are in the septa. Purple- and yellow-colored markers indicate VGluT2-labeled boutons on dendrites inside and outside the barrels, respectively. The marker shapes are classified according to the targeted neurons, that is, markers of the same shape indicate bouton contacts with dendrites originating from a common PV neuron. ***D***, Sites of gap junctions on dendrites of PV neurons reconstructed in ***C***. Gap junctions located inside and outside the barrels are marked by red and white stars, respectively. The white arrows indicate the connectivity through eight gap junctions that are found between the reconstructed PV neurons shown here. The positions of these connecting gap junctions are shown by stars of larger size, to which straight lines are drawn from the parent somata. Scale bars: ***A***, 100 µm; ***B–D***, 50 µm.

The finding that PV neurons in the ALBSF are contained in a smaller volume with a higher density than in the PMBSF raises the interesting question of whether Type 1 PV neurons, which was defined in a previous study in the PMBSF as a type of PV neuron in a barrel with all dendritic trees confined inside its home barrel (Shigematsu et al., 2019), also exist inside a much smaller barrel in the ALBSF. Serial sections cut in the coronal plane were prepared for this analysis (Fig. 7), because dendrites extending toward the upper (layer 2/3) and lower (layer 5) layers could be discriminated most easily in this plane. Two adjoining barrels were reconstructed using two neighboring sections at a rostral position close to the bregma where the ALBSF was positioned. Thirty-four PV neurons in layer 4 were traced inside or outside the two barrels (Fig. 7*C*), and they were divided into four types according to the positional relationship to the barrel boundary in the PMBSF (Shigematsu et al., 2019): Type 1 (in-barrel type), the soma and all dendrites were confined in a single barrel; Type 2 (trans-barrel type), the soma was located inside the barrel, and the dendrites crossed the barrel boundary; Type 3 (trans-septa type), the soma was located in septa, and the dendrites entered the barrel; and Type 4 (in-septa type), both the soma and the dendrites were within the interbarrel septal region. Among 17 neurons whose somata were located inside a barrel, only 1 neuron (5.9%) was found to be Type 1. The remaining 16 PV neurons (7 in the left barrel and 9 in the right barrel in Fig. 7*C,D*) had dendrites that crossed the barrel border toward the outside; thus, they were grouped into Type 2. Sixteen PV neurons belonged to Type 3 with their somata located in septa and with dendrites penetrating one or two adjoining barrels. One PV neuron was Type 4 with their somata and dendrites limited to the septa. A previous study in the PMBSF demonstrated that 17 out of 41 (41.5%) PV neurons belonged to Type 1 (Shigematsu et al., 2019). Thus, the number of Type 1 PV neurons greatly decreased in the ALBSF as compared with the PMBSF.

### Apposition of VGluT2-positive axonal boutons on PV neurons

**Figure 8.**
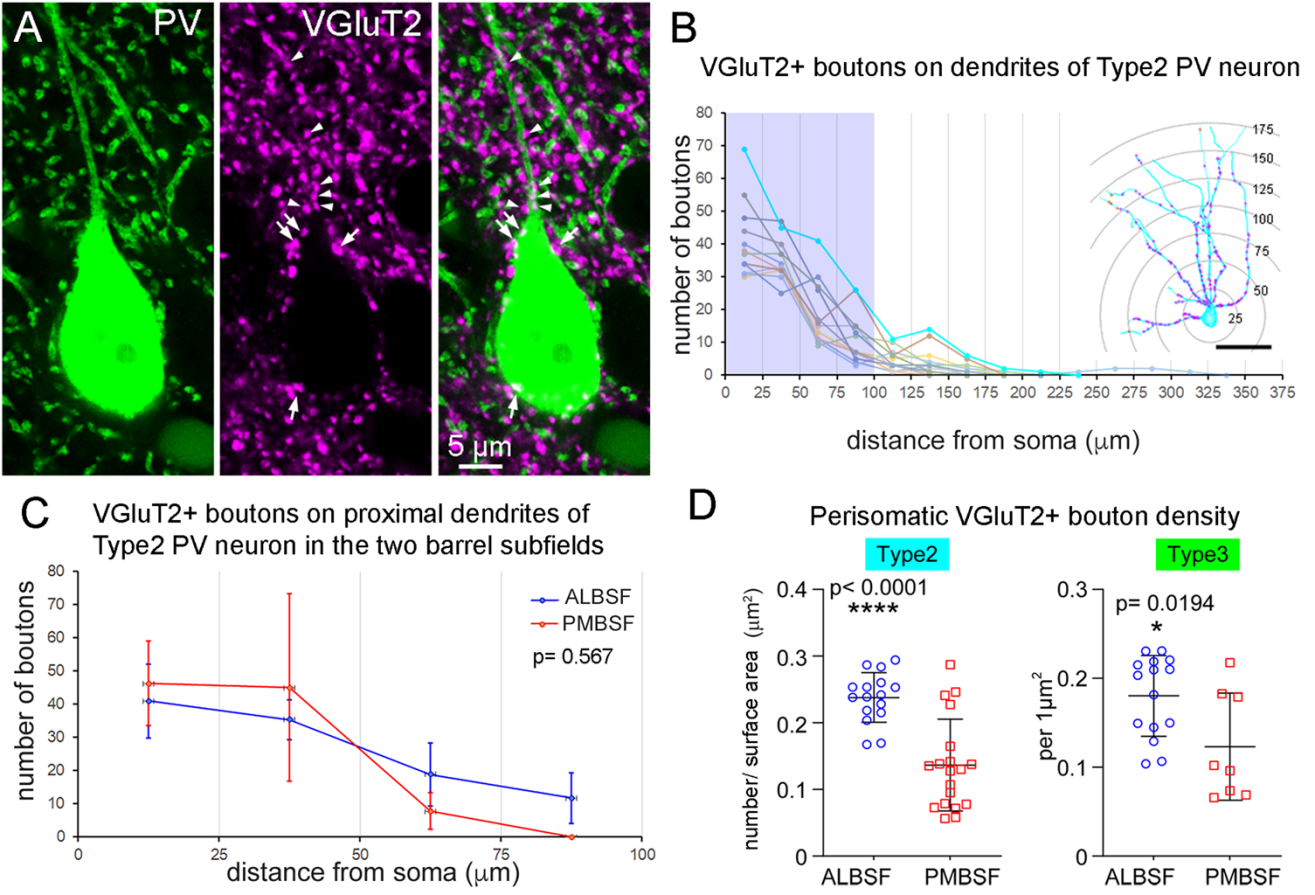
Quantitative analysis of VGluT2-positive boutons apposing on soma and dendrites of Type 2 (trans-barrel type) and Type 3 (trans-septa type) PV neurons in the ALBSF. ***A***, CLSM images showing dual immunohistochemistry for PV (green) and VGluT2 (magenta). The arrows and arrowheads in the merged image indicate the apposition of VGluT2-positive boutons on PV-positive soma and dendrite, respectively. ***B***, Line plots (left) show the number of VGluT2-positive boutons apposing on 25-μm-long segments of dendrites at different distances from soma. Data were obtained from 12 Type 2 PV neurons, shown here with different colors for individual cells. The abscissa indicates the distance from the soma. Apposition of VGluT2-positive boutons on dendrites of PV neurons decreased significantly depending on the distance from the soma (one-way ANOVA with post hoc Tukey's test: *p* < 0.0001; degree of freedom, 5; *n* = 11). The shaded area (0–100 µm) is the range of dendritic segments used for comparison with the data in the PMBSF. Inset: Reconstruction of a Type 2 PV neuron in the ALBSF with dots indicating sites of VGluT2-positive boutons on dendrites. Concentric circles are drawn in every 25 μm distance from the soma. ***C***, Comparison of the number of VGluT2-labeled boutons, which apposed on dendrites of Type 2 PV neurons at different distances from the soma, between the ALBSF and the PMBSF. There was no statistical difference between the two subfields (two-way ANOVA: *p* = 0.567; degree of freedom, 1; *n* = 11 in the ALBSF; *n* = 4 in the PMBSF). Error bars indicate SD. ***D***, Comparison of the density (number per 1 μm^2^) of VGluT2-positive boutons apposing on somata of Type 2 (left: 0.23 ± 0.04 µm^−2^; *n* = 16 in the ALBSF vs 0.14 ± 0.07 µm^−2^; *n* = 19 in the PMBSF) and Type 3 (right: 0.18 ± 0.05 µm^−2^; *n* = 15 in the ALBSF vs 0.12 ± 0.06 µm^−2^; *n* = 8 in the PMBSF) PV neurons. Statistical analysis is shown in Table 1.

During reconstructions of PV neurons in the ALBSF, we marked apposition sites of VGluT2-positive axonal boutons on dendrites and soma of PV neurons (Figs. 7*C*, 8*A*). Because VGluT2 is a marker protein to label thalamocortical glutamatergic axon terminals (Fujiyama et al., 2001), VGluT2-positive axonal boutons on PV-positive GABAergic neurons are thought to drive feedforward inhibitory circuits in layer 4. Distribution of VGluT2-positive boutons along dendrites of Type 2 (trans-barrel) and Type 3 (trans-septa) PV neurons were analyzed using the Sholl method (Fig. 8*B*). First, the dendritic tree radiating from a single neuron was segmented into 25-µm-long domains at different distances from the soma. Then, the number of VGluT2-positive boutons that made contact with radiating dendrites passing through each dendritic domain was counted and summated for each domain. It was found that Type 2 (Fig. 8*B*) and Type 3 PV neurons had a common feature that the proximal dendritic domains received more VGluT2-labeled boutons compared with the distal domains. To further explore the tendency of more contacts in the proximal positions, we statistically compared the number of VGluT2 apposition on the six dendritic domains (0–25, 25–50, 50–75, 75–100, 100–125, and 125–150 µm). Apposition of VGluT2-positive boutons on dendrites of PV neuron decreased significantly depending on the distance from the soma in both Type 2 (Fig. 8*B*) and Type 3 (one-way ANOVA with post hoc Tukey's test: *p* < 0.0001; degree of freedom 5; *n* = 12). Next, we compared the distribution patterns of VGluT2-labeled boutons on dendrites between the ALBSF and PMBSF (Fig. 8*C*). The data of the Sholl analysis in the PMBSF were acquired from a previous study (Fig. 3C in Shigematsu et al., 2019). There was no significant difference between the ALBSF and PMBSF regarding the number of VGluT2-labeled bouton appositions on proximal dendritic domains of either Type 2 (Fig. 8*C*) or Type 3 (two-way ANOVA: *p* = 0.18; degree of freedom 1; *n* = 12 in the ALBSF; *n* = 3 in the PMBSF) PV neurons.

**Figure 9.**
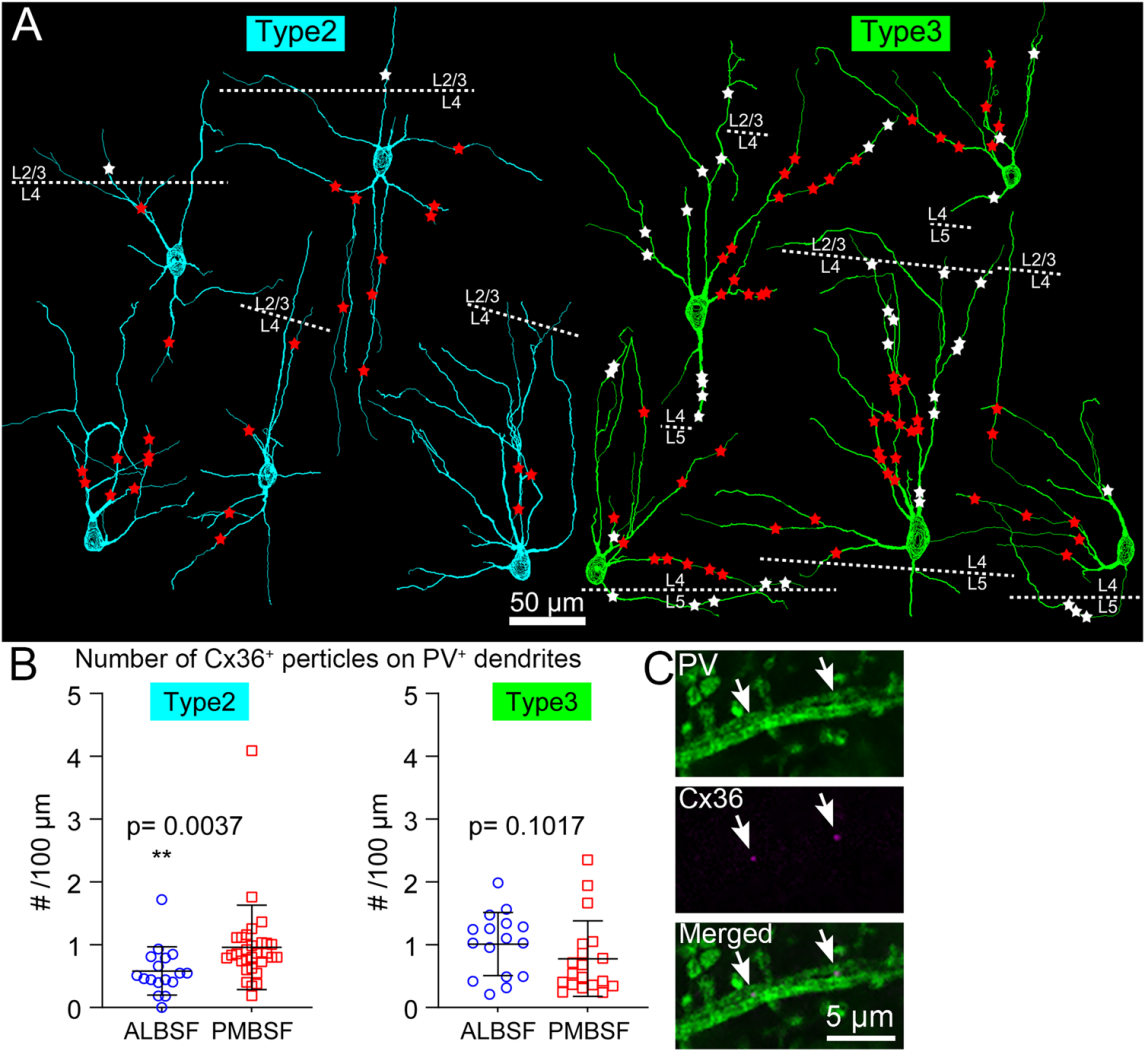
Quantitative analysis of the distribution of Cx36-positive gap junctions along dendrites of PV neurons in the ALBSF. ***A***, Reconstructions of Type 2 (left) and Type 3 (right) PV neurons contained in Figure 7*D*. Positions of Cx36-positive puncta inside and outside barrels are indicated by red and white symbols, respectively. Laminar borders are shown by dotted lines. Note that many white dots in layer 4 are located in septa. ***B***, Comparison of the density of Cx36-positive puncta (number per 100 µm) of Type 2 (left: *n* = 17 in the ALBSF; *n* = 30 in the PMBSF) and Type 3 (right: *n* = 16 in the ALBSF; *n* = 19 in the PMBSF) PV neurons between the two barrel subfields. ***C***, An example of dendrodendritic contacts of PV neurons (green) through Cx36-positive puncta (magenta). Statistical analysis is shown in Table 1.

We also counted the number of VGluT2-labeled boutons apposing on the soma of Type 2 and Type 3 PV neurons and calculated the density of bouton contacts per unit surface area of the soma (Fig. 8*D*). The data in the PMBSF were acquired from a previous study (Shigematsu et al., 2019). The densities of VGluT2-positive inputs on Type 2 and Type 3 somata in the ALBSF were 1.64 and 1.50 times higher than those in the PMBSF, respectively, with statistically significant differences (Table 1: Type 2, *p* < 0.0001 in Mann–Whitney *U* test, followed by both Bf correction and B–H procedure; Type 3, *p* = 0.0194 in Mann–Whitney *U* test, followed by both Bf correction and B–H procedure).

### Dendritic gap junctions formed between PV neurons in the ALBSF

One remarkable feature of PV neurons is that they form a network of dendrites that are electrically connected to one another through multiple gap junctions (Fukuda and Kosaka, 2000; Tamás et al., 2000; Bartos et al., 2001; Fukuda, 2017). A previous study in the PMBSF demonstrated the existence of gap junctions between dendrites of PV neurons with specialized connectivity among four different types (Shigematsu et al., 2019). In the present study, we explored whether PV neurons in the ALBSF also formed gap junctions and, if so, whether the mode of connectivity was similar to that in the PMBSF (Figs. 7*D*, 9). Sites of gap junctions were detected using immunohistochemistry for Cx36, a gap junction protein specific to neurons (Condorelli et al., 1998; Söhl et al., 1998; Rash et al., 2000; Fukuda et al., 2006).

During reconstructions of 34 PV neurons in the ALBSF (Fig. 7), Cx36-positive puncta were marked along the dendrites using Neurolucida. All these neurons were found to form gap junctions at dendrites (Fig. 9*A*). The average number of gap junctions formed by Type 2 neurons was 5.1 ± 3.7 (*n* = 16 cells) with a density of 0.58 ± 0.37 puncta per unit length (100 µm) of a dendrite, whereas that in Type 3 neurons was 9.6 ± 6.8 (*n* = 16 cells) with a density of 1.0 ± 0.49 puncta per 100 µm (Fig. 9*B*). These densities were compared with those of Type 2 (0.96 ± 0.67 puncta per 100 μm, *n* = 30 cells) and Type 3 (0.78 ± 0.60 puncta per 100 μm, *n* = 19 cells) neurons in the PMBSF; these data in the PMBSF were acquired from a previous study (Shigematsu et al., 2019). The density in Type 2 neurons in the ALBSF was significantly lower than that in the PMBSF (Table 1: *p* = 0.0037, Mann–Whitney *U* test, followed by both Bf correction and B–H procedure), whereas there was no significant difference in the density between Type 3 neurons in the ALBSF and that in the PMBSF (Table 1: *p* = 0.1017, Mann–Whitney *U* test).

Although dendrites of both Type 2 and Type 3 neurons crossed the barrel boundary, most of Cx36-positive puncta on dendrites of Type 2 neurons were located inside the home barrel, which was contrasted with the localization of Cx36-positive puncta on dendrites of Type 3 neurons both inside and outside barrels (Fig. 9*A*). This suggests more extensive involvement of Type 3 neurons in the integration of signals inside and outside their home barrels as compared with Type 2 neurons.

Regarding the type-specific connectivity, we observed eight Cx36-positive puncta (Fig. 7*D*, red stars) between the reconstructed PV neurons; the remaining Cx36-puncta along the reconstructed dendrites were made with PV-positive dendrites of unidentified origins. The connectivity was as follows: three cases in Type 2–Type 2 pairs, three cases in Type 3–Type 3 pairs, and two cases in Type 2–Type 3 pairs.

## Discussion

The present study has revealed the morphological features of ALBSF barrels for the first time. The precise quantitative analyses were performed based on a deformation-free preparation of tangential sections. In comparison with the PMBSF, the barrels in the ALBSF were characterized by their smaller size, presence of a higher density of PV neurons, paucity of Type 1 (in-barrel type) PV neurons that receive inputs exclusively inside the home barrel, and higher density of thalamocortical axonal boutons on somata of both Type 2 (trans-barrel type) and Type 3 (trans-septa type) PV neurons. All four types of PV neurons formed dendritic gap junctions with others in the ALBSF, but the density of gap junctions on dendrites of Type 2 PV neurons, which were the dominant type inside barrels in the ALBSF, was lower than that in the PMBSF. These features suggest the existence of the two vibrissa–barrel subsystems that might contribute to different animal behaviors using two types of vibrissa.

### Technical considerations

In the present quantitative analysis, we classified the barrel field of the somatosensory cortex into two subfields: PMBSF consisting of 27 posteromedial barrels and the remaining part that we simply defined as ALBSF. This does not mean that there is a strict border between the two subfields defined in this study. In fact, the size of barrels gradually became smaller when observed from the posteromedial to anterolateral direction. The tendency of gradual changes may also apply to various other features in both structure and function. Thus, the quantitative analysis in the present study might have included data in barrels showing intermediate properties, which would obscure the difference. Even so, there were significant differences in many morphological features between the two subfields. This indicates the substantial difference between the two subfields, and the difference will become more appreciable when a part of ALBSF barrels located at more distant positions from the PMBSF are selected for comparison.

According to a previous study focusing on the C2 barrel in mice, the estimated number of all neurons (sum of excitatory and inhibitory neurons) in the C2 barrel was 1,796 (Lefort et al., 2009), which is comparable to the number of NeuN-positive cells (1,704) estimated in the present analysis, even though the method for estimation is quite different between the two studies.

### Morphological differences between the two barrel subfields

The average barrel volume in the ALBSF was 34.7% of that in the PMBSF. The average number of neurons inside barrels was also smaller in the ALBSF; the number in the ALBSF was 29.0% of that in the PMBSF. Thus, information is processed by much fewer neurons in a single barrel in the ALBSF as compared with that in the PMBSF. However, this difference might not necessarily indicate the coarseness of information processed in the ALBSF. Microvibrissae are much shorter than macrovibrissae (Fig. 2), and microvibrissae do not exhibit active motion that can be observed in the mobility of macrovibrissae during whisking behavior. These differences suggest that the space scanned by a single microvibrissa is rather two-dimensional and that its size is much smaller than the space scanned three-dimensionally by a single macrovibrissa. Another feature to be considered is the difference in the density of vibrissa (number per unit skin area). The density of microvibrissae is much higher than that of macrovibrissae; the proportion of the density in the former to the latter reaches 40–100 times in rats (Brecht et al., 1997). This structural design suggests a mode of sensory detection that is executed by a large number of small-sized units, which might underlie high-resolution sensing using microvibrissae when animals discriminate fine texture (Kuruppath et al., 2014) and shape of objects (Brecht et al., 1997). In contrast, information on space such as distance and orientation will be acquired most probably through long and mobile macrovibrissae (Knutsen et al., 2006; Pammer et al., 2013; Grant et al., 2018; Huet et al., 2022). These differential properties of the two vibrissal types are thought to be reflected in morphological differences between the two barrel subfields, though it remains to be studied how functional differences in peripheral sensing apparatus result in differences that we quantified in cortical unitary structures.

The present analysis has further demonstrated significant differences in morphological features of PV neurons between the ALBSF and PMBSF. Because PV neurons have pivotal roles in regulation of cortical local circuits (Lee et al., 2012; Hu et al., 2014; Zhu et al., 2015; Garcia-Junco-Clemente et al., 2018), the differences in PV neuron morphologies may further support the dualistic view that rodents use the two vibrissa–barrel systems, macrovibrissae–PMBSF, and microbvibrissae–ALBSF, to execute qualitatively differential modes of sensory processing.

### Dendritic morphology of PV neurons

The present study focused on the morphology of soma and dendrites of PV neurons. Neurons in layer 4 of the barrel field have unique structural features in that axons and axonal boutons derived from thalamocortical lemniscal pathway concentrate inside barrels, forming clearly distinguishable patterns of barrels and septa. Principal neurons in barrels, which are termed spiny stellate cells, take a unique dendritic morphology with their skewed dendrites confined inside their home barrel. This structural design indicates that spiny stellate cells are specialized to receive thalamocortical inputs as the driving force. As to GABAergic interneurons, neurons of a similar type elongating dendrites only inside a single barrel have been repeatedly observed (Porter et al., 2001; Li and Huntsman, 2014; Koelbl et al., 2015; Shigematsu et al., 2019), and PV immunoreactivity has been reported in the majority of this neuronal type (Li and Huntsman, 2014; Koelbl et al., 2015; Shigematsu et al., 2019). In a previous study in the PMBSF, this subtype was defined as Type 1 (in-barrel type), which most likely receives excitatory driving inputs from a single vibrissa-derived lemniscal pathway [Shigematsu et al. (2019), their Fig. 7]. However, in-barrel type PV neuron was rarely observed in the ALBSF. This suggests the substantial difference between the ALBSF and PMBSF regarding the origin of driving inputs to PV neurons in barrels. The accumulation of VGluT2-positive thalamocortical axon terminals inside barrels is complementary to the distribution of VGluT1-positive corticocortical terminals that are dense outside barrels but sparse in barrels. In the ALBSF, most PV neurons inside barrels belong to Type 2 (trans-barrel type) and thus receive combined inputs originating from both peripheral sensory signals and cortical neurons. In contrast, approximately 40% of PV neurons inside barrels in the PMBSF belong to Type 1 PV neurons (Shigematsu et al., 2019), which will receive driving inputs from only peripheral signals. This difference in turn will be reflected in the differential GABAergic regulation of barrel neurons targeted by PV neurons. Therefore, the ALBSF is not just an anterolateral continuation of the PMBSF but will be involved in information processing that is qualitatively different from that in the PMBSF.

The present analysis did not consider axonal arborization patterns for the classification of PV neurons (Li and Huntsman, 2014; Koelbl et al., 2015). Thus, type-specific quantification of VGluT2 bouton density (Fig. 8*B–D*) and gap junction density (Fig. 9*B*) might be influenced by the possible mixing of different subpopulations that show different axonal morphology. In this respect, it is necessary to consider that most previous observations of layer 4 interneurons in the barrel field are conducted for those whose somata are located inside a barrel; information about septal interneurons is almost unavailable. Moreover, the present study revealed the rarity of Type 1 neurons in the ALBSF. Thus, we focus our discussion on Type 2 neurons. The preferential localization of VGluT2-positive boutons in the proximal part of Type 2 dendrites is demonstrated not only by statistical analysis but also in the curves of the graph showing a monotonous decrease in the density along the dendritic length in every cell (Fig. 8*B*), even if the graph may consist of data from different subpopulations. Therefore, it might be reasonable to conclude that dendrites of Type 2 PV neurons receive VGluT2-positive inputs preferentially on the proximal part. This is supported by the previous observations that the proximal location of thalamocortical inputs is also observable in layer 4 interneurons in the primary visual cortex (Freund et al., 1985; Fukuda, 2017). Statistical analyses further demonstrated differences in perisomatic VGluT2 bouton density (Fig. 8*D*) and gap junction density (Fig. 9*B*) between the two barrel subfields. It might be a general tendency that the mixing of subpopulations leads to obscuring differences in quantitative data. Thus, although we did not further subdivide neurons according to axonal arborization patterns, each type as a whole showed several quantitative characteristics based on statistical analyses. Future studies have to determine how different subpopulations in each type influence the results obtained in the present study.

### Thalamocortical inputs on PV neurons in the ALBSF

The present quantitative analysis revealed that VGluT2-positive thalamocortical inputs apposed preferentially on somata and proximal dendrites of PV neurons in the ALBSF. This was common to observations in the PMBSF, indicating that PV neurons in both barrel subfields are embedded in the feedforward inhibitory circuit evoked by thalamocortical inputs (Agmon and Connors, 1992; Swadlow, 2003; Cruikshank et al., 2007; Kimura et al., 2010). However, the present analysis further suggests that PV neurons in the ALBSF may mediate this feedforward regulation more powerfully than PV neurons in the PMBSF, on the basis of three quantitative data. First, the density of VGluT2-positive boutons on soma, which are thought to provide the most powerful driving inputs on a neuron, was 1.64 times higher in Type 2 PV neurons in the ALBSF as compared with those in the PMBSF. Second, the proportion of PV neuron number to the total number of all neurons inside a barrel was 1.69 times higher in the ALBSF. Third, the proportion of PV neuron number to the total number of all GABAergic neurons inside a barrel was 1.20 times higher in the ALBSF. These findings suggest possible differences in the degree of feedforward regulation, which in turn will influence the mode of information processing differently in the two subfields. Previous in vivo recordings in the rat barrel cortex revealed that feedforward inhibitory circuits mediated by FS, presumptive PV-positive neurons increase the temporal resolution of barrel neurons in layer 4 (Gabernet et al., 2005). Moreover, activation of PV neurons, but not other types of cortical GABAergic neurons, contributes to the improvement of feature selectivity and perception in the primary visual cortex in vivo (Lee et al., 2012). Although it remains to be studied how the difference in inhibitory circuitry can be associated with behavioral differences, it can at least be said that information originating from microvibrissae needs more involvement of PV neurons in the processing inside barrels as compared with macrovibrissae-derived information.

Both excitatory and inhibitory inputs are shared by pairs of PV neurons connected by gap junctions, which could lead to not only synchronous activities of coupled cells but also various electrical outcomes ranging from mutual excitation and inhibition, coincidence detection, enhancing signal-to-noise ratio, and even desynchronization (Connors, 2017; Vaughn and Haas, 2022). Despite many studies regarding detailed physiological functions of gap junctions at the cellular level in vitro, it remains almost unknown how gap junctions influence population activities in the mammalian cerebral cortex in vivo and the resultant behaviors of animals, because investigations of gap junctions at the network level in mammalian brains have been conducted mainly by morphological analysis (Fukuda, 2017; Shigematsu et al., 2019). However, the difference in the density of gap junction coupling between the two barrel subfields shown here will provide important clues for studies in computational neuroscience that incorporate numeric data of gap junctions into realistic models to simulate neuronal population activities as executed in other brain regions (Steyn-Ross et al., 2007; Zsiros et al., 2007; Stacey et al., 2009; Sun, 2009; Shinozaki et al., 2013; Amsalem et al., 2016; Steyn-Ross and Steyn-Ross, 2016).

### Septa

The present analysis has also revealed regional differences in the density of PV neurons in the septa. Neurons in the septa receive thalamic inputs from two different nuclei, namely, the POm through the paralemniscal pathway and the head region of the VPM through the lemniscal branch 2 pathway (Koralek et al., 1988; Urbain and Deschênes, 2007; Furuta et al., 2009; Wimmer et al., 2010). These pathways convey information derived from multiple whiskers. In contrast, barrel neurons receive thalamic inputs exclusively from the VPM core (lemniscal branch 1 pathway) that relays information largely from a single whisker. Thus, septal neurons will be engaged in the integration of lemniscal and paralemniscal information streams, in which a higher density of PV neurons in the septa of the ALBSF might be also associated with higher-resolution sensing using microvibrissae.

## Synthesis

Reviewing Editor: Miriam Goodman, Stanford University Decisions are customarily a result of the Reviewing Editor and the peer reviewers coming together and discussing their recommendations until a consensus is reached. When revisions are invited, a fact-based synthesis statement explaining their decision and outlining what is needed to prepare a revision will be listed below. The following reviewer(s) agreed to reveal their identity: Chris Bresee.

I am pleased to inform you that both reviewers appreciated the improvements to the manuscript during revision. I have only one small editorial request which is to covert the extended data figure4-1 to a table in the main manuscript so that readers can more readily access the statistical analysis.
